# Understanding Dyslexia in the Context of Developmental Language Disorders

**DOI:** 10.1044/2018_LSHSS-DYSLC-18-0049

**Published:** 2018-10-24

**Authors:** Suzanne M. Adlof, Tiffany P. Hogan

**Affiliations:** a University of South Carolina, Columbia; b MGH Institute of Health Professions, Boston, MA

## Abstract

**Purpose:**

The purpose of this tutorial is to discuss the language basis of dyslexia in the context of developmental language disorders (DLDs). Whereas most studies have focused on the phonological skills of children with dyslexia, we bring attention to broader language skills.

**Method:**

We conducted a focused literature review on the language basis of dyslexia from historical and theoretical perspectives with a special emphasis on the relation between dyslexia and DLD and on the development of broader language skills (e.g., vocabulary, syntax, and discourse) before and after the identification of dyslexia.

**Results:**

We present clinically relevant information on the history of dyslexia as a language-based disorder, the operational definitions used to diagnose dyslexia in research and practice, the relation between dyslexia and DLD, and the language abilities of children with dyslexia.

**Conclusions:**

We discuss 3 clinical implications for working with children with dyslexia in school settings: (a) Children with dyslexia—with and without comorbid DLDs—often have language deficits outside the phonological domain; (b) intervention should target a child's strengths and weaknesses relative to reading outcomes, regardless of diagnostic labels; and (c) those who have dyslexia, regardless of language abilities at the time of diagnosis, may be at risk for slower language acquisition across their lifetime. Longitudinal studies are needed to assess multiple language skills early, at the time of the diagnosis of dyslexia, and years later to better understand the complex development of language and reading in children with dyslexia.

Although the term *dyslexia* is familiar to most of the lay public, there is no consensus on precise diagnostic criteria. Most definitions of dyslexia agree on primary inclusionary criteria, including marked difficulties with word reading, decoding, and spelling as evidenced by low accuracy and/or fluency on standardized assessments. There is also a general agreement that these difficulties should be inconsistent with or “unexpected” in consideration of other aspects of development, including general intellectual abilities (American Psychiatric Association [APA], 2013; Lyon, Shaywitz, & Shaywitz, 2003; National Institute of Neurological Disorders and Stroke, 2017; Tunmer & Greaney, 2010). For example, children with hearing or vision impairment or with neurodevelopmental syndromes or who have had a prior head injury may experience reading and spelling difficulties as a result, but they would not be considered to have dyslexia. Some definitions further specify that poor instruction should be ruled out as a cause of reading and spelling difficulty (APA, 2013; Lyon et al., 2003). In research and practice, the operationalization of these inclusionary and exclusionary criteria varies widely, leading to sizeable variation in estimated prevalence rates—from as low as 3% to as high as 20% of the population (Rutter et al., 2004; Shaywitz, 1996; Spencer et al., 2014).

One source of confusion concerns perceptions about the oral language abilities of children with dyslexia. On the one hand, dyslexia has been described as a “language-based” disorder for many years; such descriptions have been focused primarily on phonological deficits as a core feature of dyslexia (Lyon et al., 2003; Moats, 2008). On the other hand, there is less clarity about the extent to which other aspects of language development, such as vocabulary, syntax, and discourse, are affected in individuals with dyslexia. Although one line of research has established that dyslexia and developmental language disorder^1^ (DLD; Bishop, Snowling, Thompson, Greenhalgh, & CATALISE-2 consortium, 2017) are separate disorders that frequently co-occur (Catts, Adlof, Hogan, & Weismer, 2005), some experts have suggested that the presence of DLD would make word reading difficulties no longer “unexpected” and therefore should exclude a child from the classification of dyslexic (Badian, 1999; Silliman & Berninger, 2011; Spencer et al., 2014; Tunmer & Greaney, 2010). In this article, we consider the language basis of dyslexia from a historical and theoretical perspective drawing from pertinent empirical work. We discuss the overlap of dyslexia and DLD and their relative frequency, followed by clinical implications and directions for future research.

## Defining Dyslexia as a “Language-Based” Disorder

When William Berlin first introduced the term *dyslexia* in 1887, he used it to describe adult patients who had reading problems as a result of cerebral disease, and the disorder was conceptualized within the general class of aphasias (Richardson, 1992). The first published case study of a developmental reading disorder was written by W. Pringle Morgan, who used the term *congenital word blindness*, in 1896. Morgan's description of “Percy,” a 14-year-old boy with severe reading difficulty, bears striking resemblance to the current characterizations of children with dyslexia: “He has been at school or under tutors since he was 7 years old, and the greatest efforts have been made to teach him to read, but, in spite of this laborious and persistent training, he can only with difficulty spell out words of one syllable…. I may add that the boy is bright and of average intelligence in conversation. His eyes are normal…and his eyesight is good. The schoolmaster who has taught him for some years says that he would be the smartest lad in school if the instruction were entirely oral” (Morgan, 1896). Subsequent articles by James Hinshelwood (1907, 1917) reported six cases of children with congenital word blindness across two generations of a single family, providing suggestive evidence of a genetic component that is consistent with modern-day evidence (Snowling & Melby-Lervåg, 2016). Approximately 30 years after Morgan's first case was reported, Samuel Orton examined over 1,000 children in the state of Iowa to determine the prevalence of word blindness, finding that one in 10 children had marked difficulty with reading words (Orton, 1937). Orton observed that many of these children had a history of oral language problems, and he was one of the first to frame dyslexia as part of a larger set of DLDs. Since those foundational studies, dyslexia has been referred to by many other terms such as visual agnosia for words, psycholexia, strephosymbolia, and specific reading disability (Wolf & Ashby, 2007).

Contemporary researchers have confirmed Orton and Morgan's notion of dyslexia as a language-based disorder (Elbro, Borstrøm, & Petersen, 1998; Shaywitz, 1998; Snowling, 1998), based primarily on deficits in the phonological domain. In a 1989 article entitled “Defining Dyslexia as a Language Based Disorder,” Hugh Catts stated, “Dyslexia is a developmental language disorder that involves a deficit(s) in phonological processing. This disorder manifests itself in various phonological difficulties as well as a specific reading disability” (Catts, 1989, p. 50; see also Catts, 1996; Catts & Kamhi, 1999). Explicitly labeling dyslexia as a language-based disorder was, in part, a strong and direct response to the misperception that dyslexia is a visually based disorder (cf. American Academy of Pediatrics, 2009). It is noteworthy that Hinshelwood had also presented strong arguments against a visual deficits explanation for word blindness as early as 1900 (Hinshelwood, 1900). The primary phonological deficit associated with dyslexia negatively impacts the specificity at which sounds are stored and recalled in words as well as an individual's ability to manipulate sounds in words and connect sounds to letters to read words. There is now an abundance of evidence that children with dyslexia, on average, perform poorly on tasks that involve phonology including phoneme awareness, word and nonword repetition, and word retrieval (see review by Vellutino, Fletcher, Snowling, & Scanlon, 2004).

As we have reviewed, dyslexia is defined as a difficulty with word level reading and spelling skills, which are in turn caused by phonological deficits. However, being a good reader involves more than only reading the words on a page. As conceptualized in the simple view of reading (Gough & Tunmer, 1986; see also Foorman, Petscher, & Herrera, 2018; Language and Reading Research Consortium, 2015), reading comprehension is the product of accurate and efficient word reading and language comprehension. The language comprehension component (sometimes called “linguistic comprehension” or “listening comprehension”) encompasses all of the linguistic knowledge and skills required for a listener to comprehend a text if it was read aloud, including vocabulary and semantic processing, syntax, inferencing, and discourse. In contrast to the large amount of evidence for phonological deficits in children with dyslexia, the status of their broader language abilities in these domains outside phonology is less clear. Many studies have reported that, in addition to phonological deficits, children with dyslexia also have weaknesses in other aspects of language including vocabulary, morphology, syntax, and discourse, often before the onset of formal reading instruction (e.g., Catts, Fey, Zhang, & Tomblin, 1999; Scarborough, 1990; Snowling, Gallagher, & Frith, 2003). However, two factors complicate the determination of language (dis)abilities in children with dyslexia. The first is variation in how the definition of dyslexia is operationalized for diagnosis. The second is variation in the time of onset of oral language difficulties. Noting the time of onset is important because reading difficulties can themselves cause slower language development, as much of language is learned via reading experience (Cunningham & Stanovich, 1997; Huettig, Lachmann, Reis, & Petersson, 2017).

## Operationalizing the Definition of Dyslexia

Morgan's (1896) description of Percy was the first documented case of childhood dyslexia, and it included multiple characteristics present in contemporary definitions of dyslexia (APA, 2013; Lyon et al., 2003): (a) a severe difficulty learning to read, despite (b) normal vision, (c) adequate instruction, and (d) average intelligence. Given these characteristics, as well as the boy's ability to learn from oral instruction, the reading problem is quite “unexpected” (cf. Lyon et al., 2003). However, how is this “unexpected” deficit operationalized in the diagnosis of dyslexia, and how do language skills outside the domain of phonology factor in? Although Morgan's description of Percy noted strong oral language abilities, that characteristic does not appear in most contemporary definitions of dyslexia (but see Tunmer & Greaney, 2010).

Traditionally, an IQ achievement discrepancy approach was used to operationalize dyslexia definitions for diagnosis for educational or research purposes. Under this approach, children were considered to have dyslexia when their word reading skills, as measured by norm-referenced measures of word reading speed or accuracy, were “discrepant” from their intelligence (Pennington, Gilger, Olson, & DeFries, 1992; Shaywitz, Shaywitz, Fletcher, & Escobar, 1990). Under this approach, it was assumed that the IQ score was an indicator of a child's potential, and a word reading score that fell significantly below an IQ score was viewed as evidence that the child was not performing at his or her full potential. Also under this approach, IQ was often quantified by a full-scale IQ that was a composite of both verbal and nonverbal IQ scores. Thus, under this approach, children with broad language deficits were less likely to qualify for a dyslexia diagnosis than children with normal language abilities because children with broad language deficits would be unlikely to achieve a high verbal IQ score. Instead, children with IQ scores commensurate with their word reading deficits were often referred to as “garden variety” poor readers, and it was believed that that they would not experience the same benefit from reading interventions as children with dyslexia (Gough & Tunmer, 1986; Stanovich, 1991).

The IQ achievement discrepancy model fell out of favor for several reasons. First, there were statistical issues: The size of the observed discrepancy would depend on the tests used (i.e., some word reading and IQ tests were easier than others), and because of regression to the mean (i.e., extreme scores are statistically more likely to be preceded or followed by less extreme scores), children with high IQs were more likely to qualify as dyslexic than children with low IQs (Francis et al., 2005). In addition, because reading requires formal instruction, it could take several years for test scores to suggest a “significant” discrepancy between IQ and reading achievement (Fletcher et al., 1998), often delaying access to interventions. Finally, there was a lack of evidence that reading profiles were different between discrepant and nondiscrepant poor readers (Siegel, 1989; Stanovich, 1991), and both groups were able to improve their reading skills when provided an evidence-based intervention (Vellutino, Scanlon, & Jaccard, 2003).

As an alternative to the IQ discrepancy approach, a somewhat more liberal approach to diagnosing dyslexia has been to use an IQ cutoff to rule out low cognitive abilities with no stipulation of a discrepancy between IQ and word reading abilities (Vellutino, Scanlon, & Reid Lyon, 2000; Wimmer, Mayringer, & Landerl, 2000). In practice, this meant that children with dyslexia had low word reading in the presence of “normal” intelligence. Although both verbal and nonverbal IQ scores have been used with this approach (e.g., Casalis, Leuwers, & Hilton, 2012; Zoccolotti et al., 2013), most current diagnostic criteria for dyslexia quantify adequate cognition using only nonverbal IQ measures and a liberal cutoff that does not qualify the child as being “cognitively impaired,” for example, within 2 *SD*s of the mean (e.g., Alt et al., 2017). Relative to the IQ discrepancy approach, the IQ cutoff approach provides a greater opportunity for children with language deficits beyond the domain of phonology to be identified as having dyslexia because it does not require that a child have a high verbal IQ.

As the field grappled with how to operationalize “average intelligence” in the diagnostic criteria for dyslexia, the importance of “adequate instruction” also came into the forefront. An influential study by Vellutino and colleagues (1996) focused on first-grade students with poor word reading abilities. When these children were provided one semester of high-quality, evidence-based reading instruction, the majority of them showed substantial improvement, such that they were no longer considered poor readers. The smaller group of children that did not respond to treatment showed poorer phonological skills before the onset of instruction than those who did respond. The authors recommended that only those who do not respond to high-quality, evidence-based reading instruction should be considered reading disabled, whereas the others may have demonstrated initially low reading scores due to experiential or instructional deficits. On the basis of the results of this study and others like it (Al Otaiba & Fuchs, 2002; Torgesen, 2000; Wolf, 1999), the reauthorization of the federal special education law in 2004 (Individuals with Disabilities Education Improvement Act, 2004; PL 108-446) allowed for identification of learning disabilities based on a student's failure to respond to scientifically based instruction. The diagnosis of dyslexia then became less important for public schools using this approach because it was a failure to respond to intervention, rather than a specific diagnostic label, that led to special education services. However, children meeting the standard criteria for dyslexia would still be identified for these services if they were not making adequate progress in response to evidence-based instruction in the regular education system. Research that has examined predictors of response to instruction has shown that children with broader language deficits, including problems with vocabulary and grammar, tend to show poorer responses to instruction than children whose language difficulties are restricted to phonology (Al Otaiba & Fuchs, 2006; F. J. Duff et al., 2008; Vadasy, Sanders, & Abbott, 2008; Whiteley, Smith, & Connors, 2007).

## The Relationship Between DLD and Dyslexia

To some, the characterization of dyslexia as a language-based disorder may be confusing in light of another prominent language disorder, DLD. Children with DLD have an unexpected deficit in language abilities despite adequate environmental stimulation and cognitive abilities with no neurological impairments (Bishop et al., 2017; L. B. Leonard, 2014; National Institute of Deafness and Other Communication Disorders, 2017). These children may have language deficits across multiple dimensions of language—phonology, morphology, syntax, vocabulary, and pragmatics—but operational definitions often require deficits in more than one language domain (Bishop et al., 2017; Tomblin et al., 1997). Although DLD is recognized as a persistent disorder with negative impacts on literacy, academic progress, and employment opportunities (Nippold, Mansfield, Billow, & Tomblin, 2008; Snowling, Duff, Nash, & Hulme, 2016; Whitehouse, Watt, Line, & Bishop, 2009), evidence suggests that a large proportion of children who qualify as having DLD are either not identified or are identified in later school grades, based on problems with reading comprehension (Catts, Adlof, & Weismer, 2006; Conti-Ramsden, Simkin, & Pickles, 2006; Nation, Clarke, Marshall, & Durand, 2004; Tomblin et al., 1997). It has been argued that parents and teachers may be more aware of problems with speech articulation and word reading than problems with understanding and producing oral language (Adlof, Scoggins, Brazendale, Babb, & Petscher, 2017; Catts et al., 2005; Nation et al., 2004; Silliman & Berninger, 2011).

There are clear parallels between the definitions of dyslexia and DLD. First, they both involve a deficit that is “unexpected” given the absence of intellectual disabilities, perceptual deficits, or other medical explanations for the observed deficits. Second, they both stipulate adequate environmental stimulation. In the case of dyslexia, the unexpected deficit is in word reading, and adequate stimulation is appropriate instruction in reading. In the case of DLD, the unexpected deficit is in overall language development, and adequate stimulation is human language interactions. Interestingly, there has been a recent surge of advocacy in the United States to raise awareness about dyslexia (Ward-Lonergan & Duthie, 2018), and internationally to raise awareness of DLD (Bishop, Clark, Conti-Ramsden, Norbury, & Snowling, 2012), but this advocacy is generally conducted in parallel with relatively little attention to co-occurrences.

If dyslexia is a language-based disorder, then do all children with dyslexia have DLD? Although the question appears to be straightforward, the varied criteria used to diagnose dyslexia have made answering this simple question complex. G. M. McArthur, Hogben, Edwards, Heath, and Mengler (2000) pooled study samples from prior research to examine the proportion of children receiving services for DLD or dyslexia who would meet diagnostic criteria for both disorders. They found that 55% of children with dyslexia could be classified as having DLD, and 51% of children with DLD could be classified as having dyslexia. Furthermore, all but 10% of children with dyslexia scored below average on standardized language assessments, and all but 20% of children with DLD scored below average on reading measures. These findings raised questions about whether dyslexia and DLD were different manifestations of the same disorder (Bishop & Snowling, 2004; Catts et al., 2005). Perhaps, the diagnostic label assigned to a child experiencing reading or language difficulties was simply a reflection of the practitioner assigning it (e.g., school psychologist vs. speech-language pathologist).

**Figure 1.**
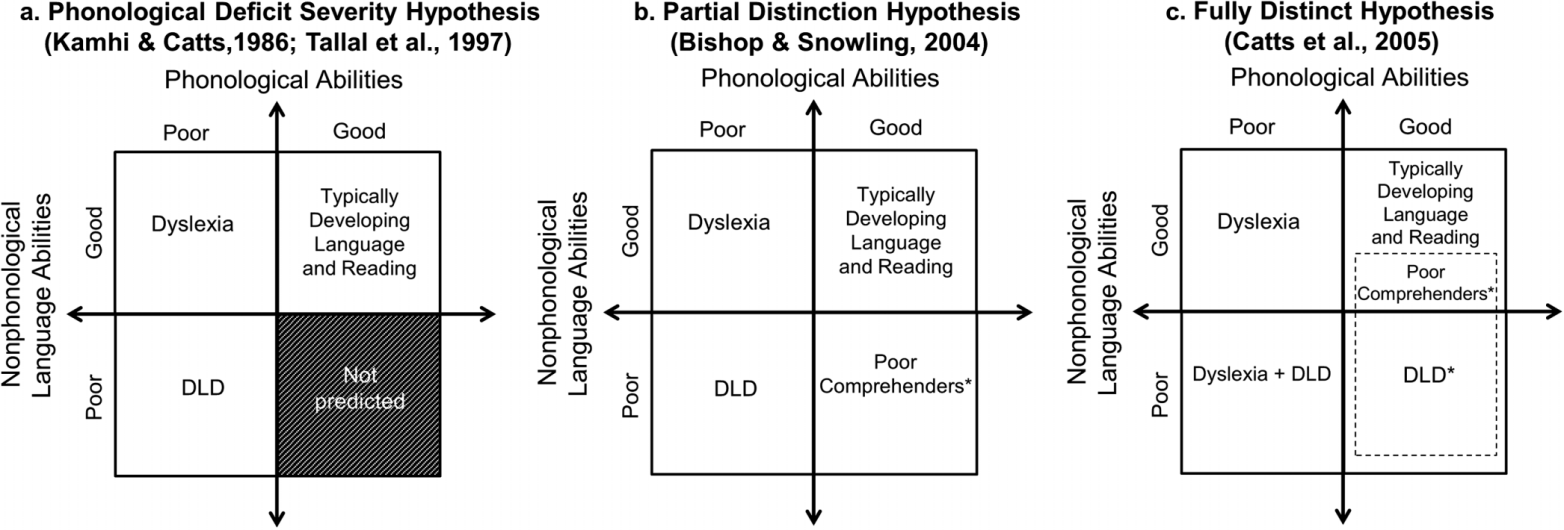
Three hypotheses about the relation between dyslexia and developmental language disorder (DLD) tested by Catts et al. (2005). Panel a depicts the phonological severity deficit hypothesis (Kamhi & Catts, 1986; Tallal, Allard, Miller, & Curtiss,1997), in which both dyslexia and DLD are caused by phonological deficits, with more severe phonological deficits leading to deficits in nonphonological domains. This hypothesis was rejected because of the existence of numerous children who showed deficits in vocabulary, grammar, and discourse, despite good skills in phonology. Panel b depicts the partial distinction hypothesis (Bishop & Snowling, 2004), in which all children with DLD show poor phonology (and therefore poor word reading), but in addition, they also have deficits in the other aspects of language, including vocabulary, grammar, and discourse. This hypothesis was rejected because of the existence of numerous children who met the standard diagnostic criteria for DLD but did not have poor phonology or poor word reading. Panel c depicts the fully distinct hypothesis, in which dyslexia and DLD are fully distinct disorders, with different underlying deficits. This model was supported by data from a large sample of children drawn from an epidemiologic study investigating the prevalence of DLD (Catts et al., 2005) and has been supported in numerous follow-up studies (e.g., Adlof et al., 2017; Bishop et al., 2009; Fraser et al., 2010; Ramus et al., 2013). Children who are referred to in studies as “poor comprehenders” display poor reading comprehension despite adequate word reading abilities. Studies indicate that approximately one third of poor comprehenders met the standard diagnostic criteria for DLD (Adlof & Catts, 2015; Catts et al., 2006; Nation et al., 2004). The remaining two thirds exhibited moderate deficits in vocabulary, syntax, and discourse, although they did not qualify as having DLD.

In a 2004 review of the literature, Bishop and Snowling proposed that a partial distinction between DLD and dyslexia should be maintained, stating, “It is important to distinguish children with relatively pure phonologically based reading problems from those with more global oral language impairments” (p. 862). They proposed a two-by-two model crossing phonological deficits against broader, nonphonological language skills (e.g., morphology, vocabulary, and syntax). As shown in Figure 1b, they hypothesized that phonological deficits underlie both dyslexia and DLD, but the two disorders would be differentiated on the basis of broader language skills. Whereas children with DLD would show deficits in both phonological and nonphonological language skills, skills outside the phonological domain would be relatively intact for children with dyslexia. Thus, in Bishop and Snowling's (2004) model, most children with DLD should have dyslexia, because of presumed underlying phonological deficits, but not all children with dyslexia would have DLD.

Catts et al. (2005) tested Bishop and Snowling's (2004) partial distinction hypothesis and two competing hypotheses, which we refer to here as the “phonological deficit severity” hypothesis and the “distinct disorders” hypothesis. The phonological deficit severity hypothesis (see Figure 1a) proposed that phonological deficits underlie both DLD and dyslexia, but these phonological deficits are more severe in children with DLD and have negative impacts on the development of broader language skills. Under the phonological deficit severity hypothesis, all children with DLD should have phonological deficits that lead to dyslexia. The distinct disorders hypothesis (see Figure 1c) posited that DLD and dyslexia are fully distinct and separate disorders that frequently co-occur, with dyslexia characterized by phonological deficits and DLD characterized by language deficits outside the phonological domain. The key difference between this hypothesis and Bishop and Snowling's (2004) partial distinction hypothesis is that the distinct disorders hypothesis predicted that some children with DLD—that is, those without dyslexia—would have phonological skills in the normal range.

Catts et al. (2005) had three important strengths present in few prior or subsequent studies. First, the study involved over 500 children who were drawn from a population-based sample who had participated in an epidemiologic study of language impairment. In contrast, most other studies have involved clinically referred samples, which likely include participants with more severe deficits and potentially more overlap between DLD and dyslexia. Second, Catts et al. (2005) assessed reading and language skills in the same children from kindergarten through eighth grade. The DLD diagnosis was determined by kindergarten language scores, and children meeting the criteria for dyslexia were identified at the second, fourth, and eighth grades. In contrast, most other studies have examined a single time point, making it difficult to disentangle language problems that may have been caused by reading difficulties. Third, Catts et al. (2005) used seven different methods to classify children as having dyslexia when examining the overlap between DLD and dyslexia: IQ discrepancy models based on (a) full-scale IQ and (b) nonverbal IQ, which did not require low achievement (such that children with word reading abilities in the normal range who still showed a discrepancy from IQ would be classified as dyslexic); IQ discrepancy models based on (c) full-scale IQ plus low achievement and (d) nonverbal IQ plus low achievement; and IQ cutoff models based on (e) full-scale IQ, (f) nonverbal IQ, and (g) low word reading without reference to intelligence.

Catts et al. (2005) found that 17%–36% of children with kindergarten DLD also met criteria for dyslexia in the second through eighth grades, depending on the criteria used to diagnose dyslexia. The lowest rates of overlap were observed when dyslexia was diagnosed using a full-scale IQ discrepancy formula (17.0%–18.8% overlap), and the highest rates of overlap were observed for the low achievement definition with no reference to IQ (31.0%–35.6% overlap). Using IQ discrepancy and low achievement criteria, 14%–19% of children with dyslexia in the second through eighth grades also met the criteria for DLD. Although the rates of overlap were significantly higher than would be expected by chance, they were considerably lower than the rates of overlap that had been reported in prior studies involving clinically referred or convenience samples. In fact, in this population-based sample, the majority of children with DLD did not have dyslexia and the majority of children with dyslexia did not have DLD.

In follow-up analyses, Catts et al. (2005) found that the vocabulary, morphology, and syntax deficits of children with DLD without dyslexia were just as severe as those of children with both DLD and dyslexia, which indicated that the phonological deficit associated with dyslexia did not translate to more severely impaired language skills in general. On the other hand, children with dyslexia, with or without DLD, consistently showed difficulty with phonologically based tasks, including phonemic awareness and nonword repetition. Taken together, these results indicated that phonological deficits were more closely associated with dyslexia than with DLD. It is notable that, in the Catts et al. sample, children with both DLD and dyslexia were more likely to have received clinical services in the primary grades, although their language skills were not more severely impaired compared with their peers with DLD without dyslexia. This finding provided additional evidence for the hypothesis that clinically referred samples overrepresent the overlap between DLD and dyslexia.

Considering the three hypotheses for the frequent overlap between children meeting criteria for DLD and dyslexia, Catts et al. (2005) concluded that the evidence best supported the distinct disorders hypothesis. The phonological deficit severity hypothesis was ruled out by the existence of numerous children with DLD without dyslexia. The fact that children with dyslexia, with or without DLD, consistently showed difficulty with phonologically based tasks, whereas those with DLD without dyslexia showed relatively mild and transient difficulties, was contrary to the predictions of Bishop and Snowling's (2004) partial distinction hypothesis.

Many subsequent studies have provided converging evidence for the existence of these distinct subgroups (Adlof et al., 2017; Alt et al., 2017; Baird, Slonims, Simonoff, & Dworzynski, 2011; Bishop, McDonald, Bird, & Hayiou-Thomas, 2009; De Groot, Van den Bos, Van der Meulen, & Minnaert, 2015; Eisenmajer, Ross, & Pratt, 2005; Fraser, Goswami, & Conti-Ramsden, 2010; Kelso, Fletcher, & Lee, 2007; Kim & Lombardino, 2013; G. McArthur & Castles, 2013; Ramus, Marshall, Rosen, & van der Lely, 2013). With the exception of Adlof et al. (2017) and Bishop et al. (2009), all studies involved clinically referred or convenience samples, and most studies involved participants from a wide age range (e.g., 7–12 or 6–16 years) measured at a single time point. Only Bishop et al. (2009) followed children longitudinally beginning in preschool, but both DLD and dyslexia determinations were made at the age of 9 years. Across these samples, children with DLD displayed a range of word reading abilities: Some children with DLD exhibited severe word reading deficits consistent with criteria for dyslexia, whereas others showed average or above-average word reading skills, similar to their typically developing peers. Likewise, children with dyslexia showed a range of language abilities with some severe enough to warrant a diagnosis of DLD.

In summary, current evidence suggests that dyslexia and DLD are distinct disorders, which frequently co-occur. The wide range of co-occurrence observed across studies (17%–71%) is likely due to sampling differences (clinically referred samples vs. those from epidemiological studies of the general population) and time point of the diagnosis of dyslexia and language impairment (at the same time or language impairment diagnosed earlier than dyslexia). Studies that draw from the general population and that diagnose DLD before formal schooling provide the strongest evidence because they avoid bias for comorbidity from clinically referred sampling and they avoid the impact of dyslexia on language skills through decreased reading experience.

## Language Abilities in Children With Dyslexia

Although research supports the conclusion that dyslexia and DLD are two separate disorders that frequently co-occur, some studies also suggest that children with dyslexia who do not have DLD may still present with relatively weak language skills compared with typically developing peers (Adlof et al., 2017; Bishop et al., 2009; Ramus et al., 2013). For example, Bishop et al. (2009) examined speech and language skills of children who met criteria for dyslexia and/or DLD at the age of 9 years. As a group, children with dyslexia who did not meet the criteria for DLD still showed significantly poorer vocabulary, sentence repetition, and syntactic comprehension than typically developing children, although their standard scores were within normal limits. However, other studies evidence a range of language skills in children with dyslexia who do not have DLD, with group means that are not significantly different from the typically developing controls (Eisenmajer et al., 2005; Fraser et al., 2010). In some studies, group means and standard deviations for children with dyslexia but not DLD suggest that many individuals display above-average standardized language scores (e.g., above the 50th percentile; Alt et al., 2017; De Groot et al., 2015; Kim & Lombardino, 2013). As discussed previously, almost all of these studies have involved clinical samples with relatively wide age ranges and have examined language and word reading abilities concurrently at a single point in time. This makes it difficult to determine whether the observed language deficits in children with dyslexia were present before the onset of reading instruction or whether they are a result of limited reading experience (see Cunningham & Stanovich, 1997; Huettig et al., 2017).

A recent study by Alt and colleagues (2017) attempted to overcome this issue by examining word learning abilities in second-grade children with dyslexia who did not have DLD. In this study, the mean Core Language standard score on the Clinical Evaluation of Language Fundamentals–Fourth Edition (Semel, Wiig, & Secord, 2004) was 99.96 (*SD* = 8.75) for the students with dyslexia, and the mean Expressive Vocabulary Test–Second Edition (Williams, 2007) standard score was slightly above average (*M* = 103, *SD* = 11). Despite their strong oral language and expressive vocabulary scores, when presented with opportunities to learn novel words, the children with dyslexia showed poor word learning compared with typically developing peers, especially apparent when learning the phonological aspects of words (i.e., their sounds and sound combinations in expressive and receptive tasks). Interestingly, they also had difficulty on a few visually based word-learning tasks, but note that all tasks involved some aspect of phonology.

### Preschool Language Abilities in Children With Dyslexia

Even in carefully controlled studies of school-aged children with dyslexia, it is difficult to determine if subpar language abilities in children with dyslexia were impacted by the phonological deficit central to dyslexia (most language tasks involve some phonology) and/or were a consequence of dyslexia (children with dyslexia read less, and reading text is an avenue for increasing language skills once children begin to read [Cunningham & Stanovich, 1997; Huettig et al., 2017]). Therefore, studies that examine broader language skills before formal reading instruction can be especially informative.

Studies of children with a family history of dyslexia are particularly useful for examining preschool language skills in children with dyslexia. As noted by Snowling and Melby-Lervåg (2016), children in these studies are recruited before they begin formal schooling, typically at birth, which allows for an examination of early language skills before receipt of reading instruction and before the impact of reading on language development. In addition, these studies avoid clinical bias because the reading outcome is not known when children are enrolled in the study. This is in contrast to a large proportion of studies that recruit children with an existing diagnosis of dyslexia, who are likely to be more severely affected. Third, these studies can be more efficient than a population-based longitudinal study because using this method yields a good number of children with dyslexia. This is because a child who has a family history of dyslexia (i.e., a parent or sibling is diagnosed with dyslexia) has approximately a 50% chance of also having dyslexia. In contrast, very large samples from the healthy population are required to include a similar number of children with dyslexia.

For the purpose of examining preschool language skills of children with dyslexia, we reviewed the studies included in Appendix B of Snowling and Melby-Lervåg's (2016) recent meta-analysis. In these studies, children with and without a family history of dyslexia were recruited and tested on cognitive–linguistic tasks before formal reading instruction and then tested again in the early school grades to determine who met criteria for dyslexia and who did not. This provides a helpful way to know which early skills were associated with having dyslexia and which were instead associated with having a family history of dyslexia. We focused specifically on the 24 studies that involved alphabetic languages; within that sample, 12 studies examined language skills outside the phonological domain and compared them between the reading outcome groups (Carroll, Mundy, & Cunningham, 2014; Elbro et al., 1998; Gallagher, Frith, & Snowling, 2000; Leppänen et al., 2010; Plakas, van Zuijen, van Leeuwen, Thomson, & van der Leij, 2013; Scarborough, 1990, 1991; A. Smith, Smith, Locke, & Bennett, 2008; S. L. Smith, 2009; S. L. Smith, Roberts, Locke, & Tozer, 2010; Snowling, Muter, & Carroll, 2007; Torppa, Lyytinen, Erskine, Eklund, & Lyytinen, 2010; van Bergen et al., 2011). We highlight four key findings, the first two of which are also provided in Snowling and Melby-Lervåg's meta-analysis. First, on average, children with a family history of dyslexia showed early and persistent deficits in phonology compared with their peers with no family history, but not all of them developed dyslexia (Snowling & Melby-Lervåg, 2016). Second, as a group, children with a family history of dyslexia who developed dyslexia were more severely impaired in the phonological domain of language and in broader language domains (e.g., vocabulary, grammar) compared with their peers with and without a family history who did not develop dyslexia (e.g., Carroll et al., 2014; Gallagher et al., 2000; Plakas et al., 2013; Scarborough, 1990, 1991; Snowling et al., 2007; Torppa et al., 2010). Third, in comparison with the numerous tasks used to obtain detailed profiles of skills in the phonological domain of language (e.g., Elbro et al., 1998; Leppänen et al., 2010; Plakas et al., 2013; S. L. Smith et al., 2010), relatively few tasks were used to measure broader language skills in most individual studies (but see, e.g., Snowling et al., 2007; Torppa et al., 2010). Across studies, receptive vocabulary was the most commonly studied nonphonological language task. Fourth, no studies considered whether and/or what proportion of children who did go on to have dyslexia also had comorbid DLD, and only two studies assessed broader language skills (using measures of sentence recall and vocabulary) at the time of the dyslexia diagnosis (Carroll et al., 2014; Snowling et al., 2007). Both of those studies provided evidence that those with a family history of dyslexia who went on to have dyslexia had poorer broader language skills than their peers with a family history who did not go on to have dyslexia. However, judging from the small effect sizes that represent the mean differences between groups with and without dyslexia on language measures administered before and at the time of the dyslexia diagnosis, it is likely that some but not all children with dyslexia would also qualify as having a DLD.

A final note is that few studies directly compared children with dyslexia who had a family history of dyslexia with children with dyslexia without a family history of dyslexia. Carroll et al. (2014) caution against assuming that all dyslexic children—with and without a family history—are the same. Future studies could further clarify the complex relationship between language development and dyslexia by including children with dyslexia sampled both from families with known history and from the general population and using multiple measures of language, including phonological and broader language tasks both before and at the time of the dyslexia diagnosis.

## Clinical Implications

In light of the surge in advocacy surrounding dyslexia and DLD (see Bishop et al., 2012; Bishop et al., 2017; Ward-Lonergan & Duthie, 2018), it is important that researchers, practitioners, and the public are aware that dyslexia and DLD are distinct but often co-occurring disorders. Although the exact rates of co-occurrence will depend on the specific diagnostic criteria used for both dyslexia and DLD, it is likely that at least half of the children who are identified with reading disabilities in schools or clinics will have co-occurring DLD (G. M. McArthur et al., 2000). In addition, many children with dyslexia who perform within normal limits on standardized language assessments may have subclinical language deficits that warrant monitoring and educational accommodations. As described in the next section, there are numerous questions that remain to be answered by future research. Despite these questions, the evidence we have reviewed points to several important clinical implications for individuals in school settings.

First, although many SLPs are aware that children on their caseloads may have reading difficulties, they (and other special education providers) may not be fully aware that children with identified dyslexia (or a specific reading disability) often have language needs outside the phonological domain. Children with dyslexia, by definition, have difficulties with word reading. However, as we have reviewed, many children with dyslexia will also struggle with other aspects of language that affect reading comprehension (likewise, children with DLD, by definition, struggle with language comprehension; many also struggle with word reading, and most will struggle with reading comprehension; see Figure 1c). Current assessment frameworks that are used to determine whether a child meets diagnostic criteria for dyslexia and related special education services in the US public schools do not explicitly require that oral language skills beyond phonological awareness be assessed. It is important for SLPs and other school personnel to advocate for the assessment of language skills across multiple domains during the evaluation process and for those skills to be monitored over time. Assessing multiple domains of language would include assessment of phonology, orthography, morphology, semantics, syntax, and discourse processing. Ideally, a thorough investigation of each domain would include both receptive and expressive tasks.

Second, regardless of the specific diagnostic label, intervention should target a child's strengths and weaknesses across all domains of language because they all impact reading comprehension. It is beyond the scope of this article to discuss specific intervention approaches, but we point readers to other sources that recommend and describe evidence-based instruction that explicitly and systematically teaches children phonological awareness, sound–letter associations, orthographic patterns, morphological awareness, vocabulary, syntactic awareness, and narrative and expository text structures (e.g., Al Otaiba, Rouse, & Baker, 2018; Foorman et al., 2016; Gersten et al., 2008). Collaboration between multiple service providers, including classroom teachers, speech-language pathologists, reading specialists, and other special educators, can help ensure that these domains are effectively addressed for all students (Archibald, 2017; Foorman, Arndt, & Crawford, 2011). Interprofessional education may be helpful for facilitating a successful collaboration between these varied service providers in addressing students' language and literacy needs (Wilson, McNeill, & Gillon, 2015).

Third, those who have dyslexia, regardless of language abilities at the time of diagnosis, are at risk for slower language acquisition and slower growth of world knowledge across their lifetime, as a result of reduced reading experience, a phenomenon known as the *Matthew effect*. To a large extent, the vocabulary, complex syntax, and general world knowledge that are acquired by adolescents and adults are acquired from texts (Cunningham & Stanovich, 1997; Huettig et al., 2017). The most important line of defense to prevent Matthew effects is to provide high-quality, evidence-based reading intervention as early as possible. However, compensatory techniques that build the child's exposure to rich text and create opportunities to acquire world knowledge may also help to mitigate the risk of Matthew effects (see Rappolt-Schlichtmann, Boucher, & Evans, 2018). For example, students can be encouraged to listen to audiobooks, which provide exposure to the same advanced language structures without the requirement of the child to do the heavy lifting of decoding. Milani, Lorusso, and Molteni (2010) found that children with dyslexia who were provided audiobook versions of their school textbooks showed a significant improvement in reading skills and a significant reduction in emotional or behavioral problems (as measured by parent report) over a 5-month period, relative to a control group who received only printed texts. The authors hypothesized that the audiobooks may have enhanced students' independence, therefore leading to the reduction in emotional and behavioral problems. In addition to compensatory techniques such as audiobooks, educators can also cultivate a lifelong love for reading and learning by helping children find books that match their interests and expand their knowledge of the world around them.

## Directions for Future Research

Studies of children with a family history of dyslexia suggest that more severe oral language deficits in the preschool years are associated with a higher likelihood of having dyslexia in the school grades (Snowling & Melby-Lervåg, 2016). However, on the basis of the family history studies we reviewed, which are quite comprehensive longitudinal studies of language and dyslexia, it remains unclear to what extent that early oral language deficits persisted in the school grades in children with dyslexia. We hypothesize that deficits in broader language skills such as vocabulary, morphology, and syntax may show peaks and valleys during development (cf. Scarborough, 2009) in children with dyslexia, depending on the time of assessment. Mild language deficits may appear to be remediated or compensated in the early school years as children benefit from high-quality oral language input with the onset of schooling. In later school grades, when more vocabulary and complex syntactic structures are acquired through reading experience, children with dyslexia may show Matthew effects, in which broader language skills show slower growth compared with peers without dyslexia due to less reading experience (D. Duff, Tomblin, & Catts, 2015; Pfost, Hattie, Dörfler, & Artelt, 2014; Snowling et al., 2007). Testing this hypothesis will require a longitudinal study that assesses multiple language skills early, at the time of the diagnosis of dyslexia, and years later.

In addition to the need for longitudinal studies that track language development across multiple domains before, during, and after the onset of dyslexia, there is also a need for more research to understand the mechanisms by which dyslexia and DLD manifest both separately and together in specific children. There is clear evidence that both genetic and environmental factors contribute to these disorders (Pennington & Olson, 2005; Rice, 2013) and that the neurobiological profiles of dyslexia and DLD are different (C. Leonard et al., 2002). There is also some evidence that different genetic components may be involved in dyslexia than DLD (Bishop, Adams, & Norbury, 2006). However, it is still the case that studies more frequently ignore the co-occurrence of dyslexia and DLD than account for it in their design or analyses. Accounting for this co-occurrence is of pivotal importance, so that the conclusions drawn about one disorder are not confounded by the unknown presence of the other disorder in the participant sample. There is also a need to attend more closely to factors that contribute to risk and resilience for students with dyslexia and/or DLD (Haft, Myers, & Hoeft, 2016; Rappolt-Schlichtmann, Boucher, & Evans, 2018), including but not limited to malleable environmental factors such as child–caregiver interactions around language and literacy and school instructional practices.

## Conclusions

In this article, we presented three clinical implications for working with children dyslexia in school settings: (a) Children with dyslexia—with and without comorbid DLDs—often have language deficits outside the phonological domain (in addition to core deficits in the phonological domain); (b) intervention should target a child's strengths and weaknesses relative to reading outcomes, regardless of diagnostic labels; and (c) those who have dyslexia, regardless of language abilities at the time of diagnosis, may be at risk for slower language acquisition across their lifetime. Future studies should follow the children at risk for dyslexia over time to assess multiple language skills early, at the time of the diagnosis of dyslexia, and years later to better understand the complex development of language and reading in children with dyslexia.

## Footnote

1 Recently, there has been a movement to raise awareness about developmental language disorders, in part by promoting a common terminology (Bishop et al., 2017). Before this movement, the most commonly used term in research was *specific language impairment* (SLI). All children with SLI qualify as having DLD, but the reverse is not true, as the criterion for normal nonverbal cognitive skills is stricter for children with SLI than children with DLD. In this article, we use the term DLD, although many cited studies involved children with SLI.
